# Unraveling the expression of the oncogene *YAP1*, a Wnt/beta-catenin target, in adrenocortical tumors and its association with poor outcome in pediatric patients

**DOI:** 10.18632/oncotarget.12382

**Published:** 2016-10-01

**Authors:** Rafael H. Abduch, Ana Carolina Bueno, Leticia F. Leal, Marcelo M. Cavalcanti, Débora C. Gomes, Silvia R. Brandalise, Maria J. Masterallo, José A. Yunes, Carlos E. Martinelli, Luiz G. Tone, Silvio Tucci, Carlos A.F. Molina, Fernando S. Ramalho, Ayrton C. Moreira, Izilda A. Cardinalli, Carlos A. Scrideli, Leandra N.Z. Ramalho, Margaret de Castro, Sonir R. Antonini

**Affiliations:** ^1^ Department of Pediatrics, Ribeirao Preto Medical School, University of Sao Paulo, Ribeirao Preto, Brazil; ^2^ Department of Internal Medicine, Ribeirao Preto Medical School, University of Sao Paulo, Ribeirao Preto, Brazil; ^3^ Department of Surgery, Ribeirao Preto Medical School, University of Sao Paulo, Ribeirao Preto, Brazil; ^4^ Department of Pathology, Ribeirao Preto Medical School, University of Sao Paulo, Ribeirao Preto, Brazil; ^5^ Boldrini Children's Center, Campinas, Brazil; ^6^ Federal University of Uberlandia, Brazil; ^#^Molecular Oncology Research Center, Barretos Cancer Hospital, Barretos, SP, Brazil

**Keywords:** adrenocortical tumor, outcome, YAP1, Hippo pathway, Wnt/beta-catenin pathway

## Abstract

**Background:**

Overexpression of the oncogene yes-associated-protein-1 (YAP1) is associated with increased cell proliferation in human cancers. YAP1 is a potential target of the Wnt/beta-catenin pathway, which plays an important role in adrenocortical tumors (ACT). The role of YAP1 in adrenocortical tumorigenesis has not been assessed.

**Aims:** To evaluate YAP1 expression in normal adrenals and pediatric ACT and its association with disease outcome. To investigate the interaction between *YAP1* and the Wnt/beta-catenin pathway in adrenocortical cells.

**Results:**

Strong YAP1 staining was present in fetal adrenals and pediatric ACT but weak in postnatal adrenals. In pediatric ACT, *YAP1* mRNA overexpression was associated with death, recurrent/metastatic disease and lower overall survival. The inhibition of the Wnt/beta-catenin pathway increased *YAP1* mRNA expression. *siYAP1* increased *CTNNB1*/beta-catenin expression and nuclear staining regardless of *DLV2*, moreover, it decreased cell growth and impaired cell migration.

**Materials and Methods:**

We assessed in 42 pediatric ACT samples the YAP1 protein expression by immunohistochemistry and mRNA expression by RT-qPCR and analyzed their association with outcome. As controls, we resort 32 fetal and postnatal normal adrenals for IHC and 10 normal adrenal cortices for RT-qPCR. The interaction between YAP1 and the Wnt/beta-catenin pathway was assessed in NCI-H295 adrenocortical cells by inhibiting the TCF/beta-catenin complex and by knocking down *YAP1*.

**Conclusion:**

YAP1 overexpression is a marker of poor prognosis for pediatric patients with ACT. In adrenocortical cells, there is a close crosstalk between YAP1 and Wnt/beta-catenin. These data open the possibility of future molecular therapies targeting Hippo/YAP1 signaling to treat advanced ACT.

## INTRODUCTION

Several genetic abnormalities have been found in adrenocortical tumors (ACT), the most prominent being IGF2 overexpression, *TP53* mutations and Wnt/beta-catenin abnormal signaling in both adult and pediatric ACT [1–7]. Although the understanding of ACT pathogenesis has increased over the last decades and significant progress has been achieved regarding the molecular mechanisms of adrenocortical tumorigenesis, few prognostic molecular markers have been identified for pediatric ACT to date. Irrespective of histopathological features, pediatric ACT behave differently and survival rate is higher in pediatric than in adult patients, suggesting the presence of distinct molecular abnormalities [8, 9]. Furthermore, adjuvant therapeutic options for both pediatric and adult patients with adrenocortical cancer (ACC) remain very limited [10]. Effective molecular targeted therapies have been successfully developed for various types of cancers but not for ACC.

Yes-associated protein 1 (YAP1) is a transcription factor-like protein, which belongs to the Hippo pathway. YAP1 plays an important role in growth control and organ renewal [11–13]. The *YAP1* gene is overexpressed in some of the most frequent human tumors such as colon, lung and ovarian cancers [14]. In addition, YAP1 overexpression is associated with a poor prognosis in medulloblastomas, colon and ovarian cancer [11, 15, 16]. *In vitro*, YAP1 nuclear accumulation resulted in worsening of the malignant phenotype and induced chemotherapy resistance in ovarian cancer cell lines [16]. Besides the Hippo pathway, YAP1 can also interact with additional signaling pathways such as Sonic Hedgehog (SHH), Notch and the Wnt/beta-catenin [12, 15, 17]. YAP1 cooperates with beta-catenin to activate genes involved in stem cell proliferation for epithelial repair [11]. Additionally, YAP1 has a key role in the control of subcellular localization of Dishvelled 2 (DVL2), an important component of the Wnt/beta-catenin pathway. In addition to YAP1 stimulation by the Wnt/beta-catenin pathway, YAP1 may also have an inhibitory role in Wnt/beta-catenin signaling by DVL2 blocking [17–19].

There are no studies investigating the role of YAP1 in adrenal development and tumorigenesis. For this reason, herein we investigated YAP1 expression in fetal adrenals and in postnatal normal adrenal cortices as well as in pediatric ACT and its relationship with disease stage and outcome. Moreover, we evaluated *in vitro* the association between the Wnt/beta-catenin pathway and YAP1 in adrenocortical carcinoma cells.

## RESULTS

### Clinical findings, follow-up and outcome

We studied 42 children with ACT, being 34 girls (80.9%) and 8 boys and median age at diagnosis of 31 months (5 to 186 months). Median follow-up was 3.5 years (0.3 - 17.6) and 19% of the patients died. All patients had hormone-secreting tumors: 30 (71%) mixed cortisol and androgen-secreting tumors, 11 (26%) androgen-secreting tumors, and 1 (2%) cortisol-secreting tumor. Most of the clinical and laboratory data of this cohort had been previously described [5, 20].

### *Ex vivo* study

#### YAP1 is highly expressed in fetal adrenals and in pediatric ACT but not in postnatal normal adrenals (Figure 1)

**Figure 1:**
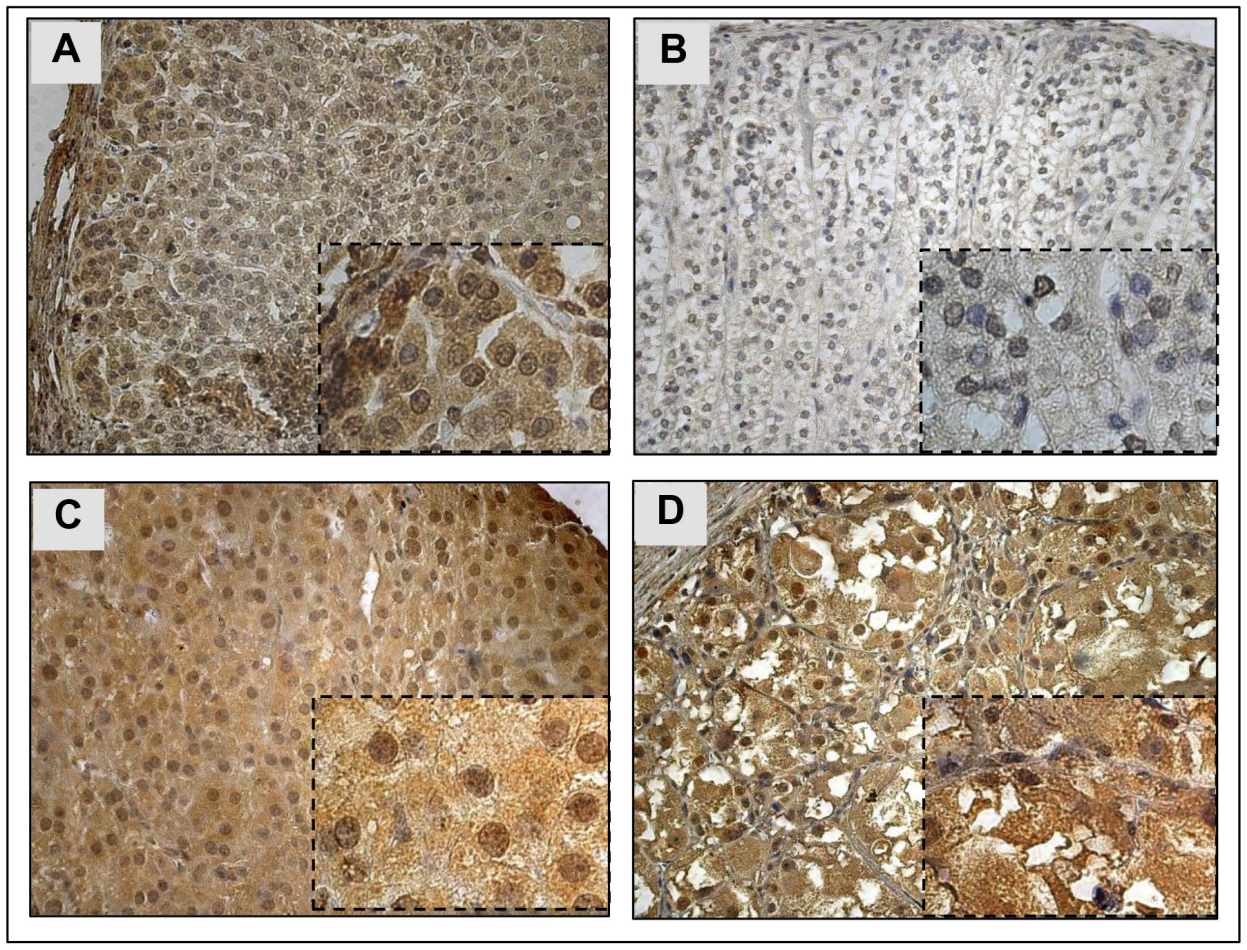
YAP1 protein* expression and localization in normal adrenal cortex and pediatric ACT **A.** YAP1 cytoplasmic and nuclear staining in normal fetal adrenal. **B.** Weak YAP1 staining in normal post-natal adrenal cortex. **C.** YAP1 cytoplasmic and nuclear staining in pediatric ACT. **D.** YAP1 cytoplasmic staining in pediatric ACT. * Mouse anti-YAP1 antibody, dilution 1:50, ab56701, Abcam. Magnification: 400x and 1000x.

In order to evaluate the role of YAP1 in adrenal development, we investigated YAP1 protein expression in fetal and postnatal normal adrenals. Fetal adrenals showed strong YAP1 nuclear staining (Figure 1A), whereas postnatal normal adrenals showed weak YAP1 staining (Figure 1B).

In pediatric ACT, YAP1 nuclear and/or cytoplasmic protein expression was observed in 85.7% (30/35) (Figure 1C–1D). Among the YAP1 positive ACT, 23.3% (7/30; all cytoplasmic) presented with moderated staining and 76.7% (23/30; all nuclear or cytoplasmic and nuclear) presented strong YAP1 staining.

#### Increased mRNA expression of YAP1 was associated with poor outcome and decreased overall survival in pediatric ACT. (Figure 2)

**Figure 2:**
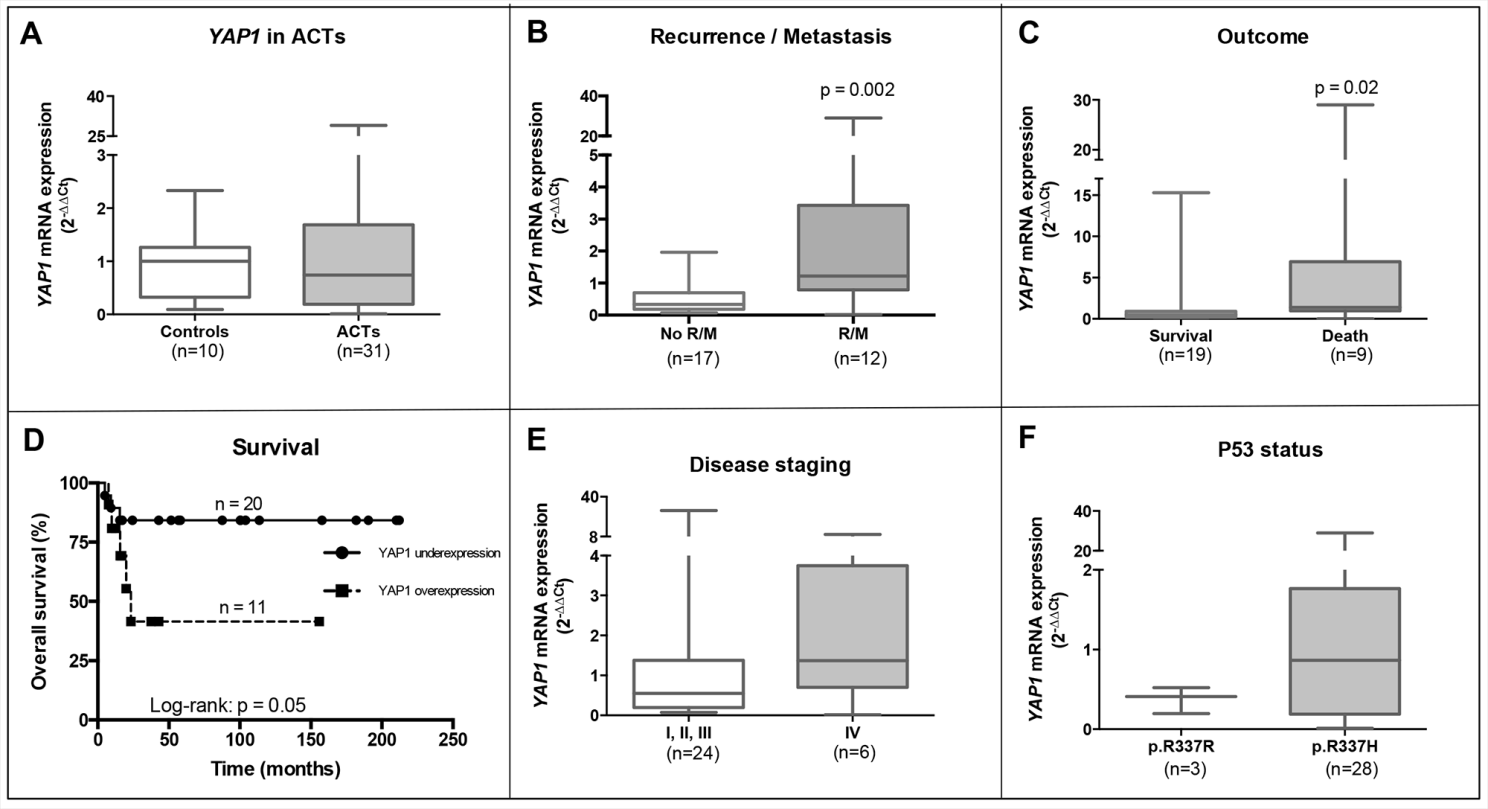
YAP1 mRNA overexpression associates with poor outcome in pediatric ACT **A.** *YAP1* mRNA is not differentially expressed in normal adrenal samples (controls) and pediatric ACT (Mann-Whitney test: p=0.99). Increased *YAP1* mRNA expression is associated with **B.** recurrent and/or metastatic disease (Mann-Whitney test: p=0.002), **C.** death (Mann-Whitney test: p=0.02) and **D.** lower overall survival (Log-rank test: p=0.05) in pediatric ACT patients. *YAP1* mRNA expression is not associated with **E.** tumor staging (Mann-Whitney test: p=0.3) and **F.** P53 p.R337H mutation (Mann-Whitney test: p=0.35) in pediatric ACT patients.

Pediatric ACT showed no differential *YAP1* mRNA expression compared with normal adrenal controls (Mann-Whitney test: p=0.99; Figure 2A), but increased *YAP1* mRNA expression was associated with recurrent and/or metastatic (R/M) disease (Mann-Whitney test: p=0.002; Figure 2B) and death (Mann-Whitney test: p=0.02; Figure 2B). In addition, *YAP1* overexpression was associated with lower overall survival of ACT patients (Log-rank test: p=0.05; Figure 2D). Regarding tumor staging according to Sandrini's classification, *YAP1* mRNA expression was not associated with advanced disease stage (Mann-Whitney test: p=0.3; Figure 2E). Regarding *TP53* status, ACT harboring the P53 p.R337H mutation showed no differential expression of *YAP1* when compared with tumors that do not harbor this mutation (Mann-Whitney test: p=0.35; Figure 2F).

Resorting to Bayesian analysis, we confirmed the association between *YAP1* mRNA expression and poor outcome as an independent variable in pediatric ACT. Increased *YAP1* mRNA expression was associated with death (Estimation: 5.09; 95%CrI: 2.09 – 8.16; Table 1) and recurrence of the disease (Estimation: 5.02; 95%CrI: 2.09 – 7.94; Table 1). In addition to poor outcome, *YAP1* overexpression was associated with advanced disease stage according to Sandrini's classification in pediatric ACT (Estimation: 4.63; 95%CrI: 0.61 - 8.64; Table 1). These results were independent of the P53 p.R337H mutation. ACT harboring *CTNNB1* mutations were not included in Bayesian analysis and *YAP1* was analyzed as an independent factor.

**Table 1:** Association between *YAP1* mRNA expression and clinical outcomes in pediatric patients with adrenocortical tumors: linear regression results based on a Bayesian model for pediatric ACT cohort

Variable	Estimation	95%CrI	
		Lower Limit	Upper Limit
Group	0.34	-2.8	3.5
**Death**	**5.09**	**2.09**	**8.16**
**Recurrence**	**5.02**	**2.09**	**7.94**
Histology	1.47	-2.12	5.06
Weiss	-1.48	-5.16	2.13
Disease stage*	**4.63**	**0.61**	**8.64**
P53 p.R337H-positive	2.76	-4.18	6.83

* Sandrini classification. Values highlighted in bold are significant (95%CrI do not contain zero between limits). 95%CrI, 95% credible interval.

### *In vitro* study

#### Inhibition of the Wnt/beta-catenin pathway impairs YAP1 expression (Figure 3)

**Figure 3:**
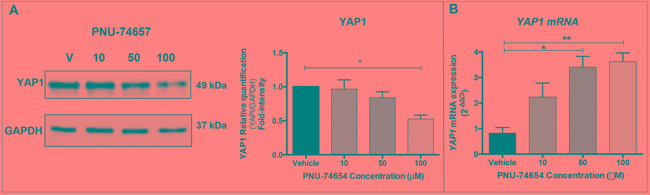
Inhibition of the Wnt/beta-catenin signaling by PNU-74654 impairs YAP1 expression in NCI-H295 ACC cell line **A.** PNU-74654 treatment decreased YAP1 protein expression in NCI-H295 cells as shown by Western blot. Fold intensity normalized by the loading control (GAPDH) is reported in the graph (Students t test: *p=0.01). **B.** PNU-74654 treatment increased *YAP1* mRNA expression in NCI-H295 cells. Values are reported as mean ± SEM. Students t test: *p=0.0035 and **p=0.0019.

We recently reported that PNU-74654 inhibited the Wnt/beta-catenin signaling by impairment of the beta-catenin expression at mRNA and protein levels, including dose-dependent decrease of the nuclear beta-catenin in NCI-H295 ACC cells [21].

Herein, we investigated whether YAP1 expression would be affected by the *in vitro* inhibition of the Wnt/beta-catenin signaling through beta-catenin decrease. We observed decreased YAP1 protein expression 48 hours after treatment with 100 μM PNU-74654 (one-way ANOVA: p=0.01; Figure 3A). Additionally, we observed increased *YAP1* mRNA expression 48 hours after treatment with 50 μM and 100 μM PNU-74654 (One-way ANOVA: p=0.0035 and p=0.0019, respectively; Figure 3B).

The half-maximal inhibitory concentration (IC_50_) of PNU-74654 for NCI-H295 cells 48 hours after treatment as well as the effect of PNU-74654 on cell viability, apoptosis and steroidogenesis had been previously described by our group [21].

#### YAP1 silencing increases CTNNB1/Beta-Catenin regardless of DVL2 mRNA expression (Figure 4A–4C)

**Figure 4:**
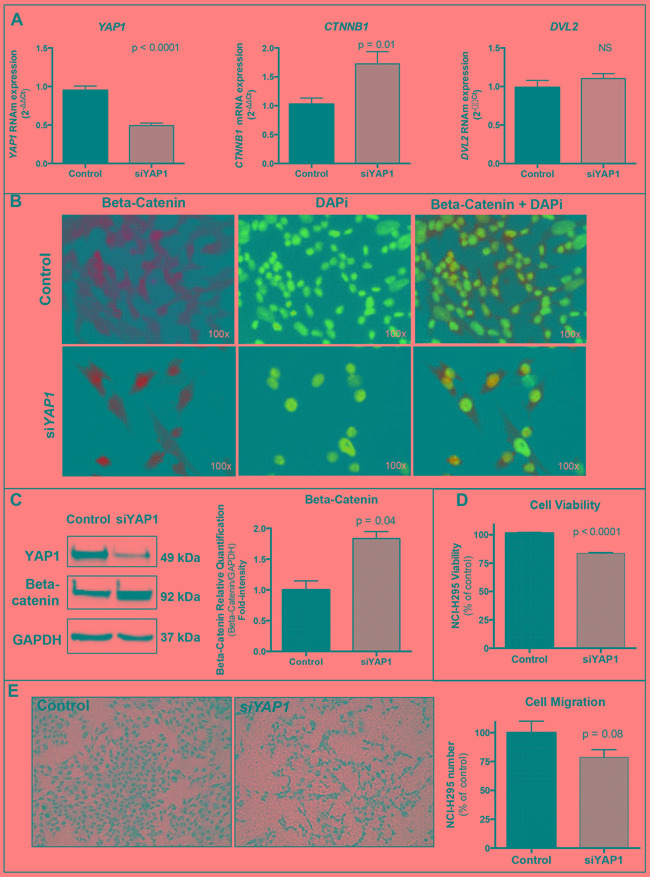
Silencing of YAP1 in NCI-H295 ACC cell line **A.** *YAP1* knockdown (Students t test: p<0.0001) increased *CTNNB1* expression (Students t test: p=0.01) regardless of *DVL2* mRNA expression (p=0.32). **B.** Immunofluorescence demonstrated increased beta-catenin nuclear expression 48 hours after silencing of *YAP1*. Green: beta-catenin (mouse anti-beta-catenin 1:2000, BD Biosciences); blue: nuclear staining (DAPi 1:25000, Cell Signaling Technology); light green: beta-catenin nuclear staining (merged). **C.** Western blot analysis confirmed YAP1 knockdown and increased beta-catenin (Students t test: p=0.04) 72 hours after silencing of *YAP1*. Beta-catenin fold intensity normalized by the loading control (GAPDH) is reported in the graph. **D.** *YAP1* silencing decreased cell viability after 96 hours (Students t test: p<0.0001). **E.** *YAP1* silencing impaired cell migration after 72 hours (Students t test: p=0.08). Values are reported as mean ± SEM.

To further investigate the interaction between YAP1 and Wnt/beta-catenin pathway, we knocked down the *YAP1* mRNA using siRNA. A fifty percent reduction in *YAP1* (Students t test: p<0.0001; Figure 4A) resulted in a 50% increment in *CTNNB1* expression (Students t test: p=0.01; Figure 4A), regardless of *DVL2* mRNA expression (Students t test: p=0.32; Figure 4A). Immunofluorescence highlighted strong beta-catenin nuclear expression after *YAP1* silencing (Figure 4B), and after 72 hours, western blot confirmed a significant 80% increase of total beta-catenin (Students t test: p=0.04; Figure 4C).

#### YAP1 silencing impairs cell migration and cell viability (Figure 4D–4E)

In order to assess the role of YAP1 in ACC cell growth and malignancy, we performed transwell migration assays. After 72 hours of *YAP1* knockdown, the cells were prone to impaired migration (-20%; Students t test: p=0.08; Figure 4E). In addition, after 96 hours of *YAP1* knockdown, cell viability was significantly decreased (-18%; Students t test: p<0.0001; Figure 4D).

## DISCUSSION

The oncogene *YAP1* plays a role in tissue renewal, cell proliferation and embryogenesis [11–13, 22]. The overexpression of YAP1 is associated with tumor progression in cancer cell lines [23]. Furthermore, YAP1 is overexpressed in common human malignancies such as colon, lung, ovary, esophagus and bladder cancer [14, 16, 22, 24]. In the present study, we evaluated, the expression of the oncogene *YAP1* in pediatric ACT as well as in fetal and postnatal normal adrenal cortices at the mRNA and protein levels.

Immunohistochemistry revealed strong YAP1 nuclear staining in fetal adrenals, whereas postnatal normal adrenals showed weak YAP1 staining. Similar to fetal adrenals, strong YAP1 staining was observed in pediatric ACT. The strong YAP1 accumulation in both nucleus and cytoplasm of fetal adrenal cells, together with decreased YAP1 expression in postnatal adrenal cortices, reinforces the role of YAP1 in promoting cell dedifferentiation and proliferation [12, 13].

Pediatric patients with ACT who experienced tumor recurrence and/or metastasis and those who died presented increased *YAP1* mRNA expression in tumor tissues. Therefore, *YAP1* overexpression in tumors was associated with lower survival in pediatric patients, reinforcing the association between YAP1 overexpression and worse prognosis. Similar to our findings, a meta-analysis study [25] confirmed that YAP1 overexpression was associated with lower overall survival and lower disease-free survival in other cancers. An association between worse prognosis and YAP1 overexpression has been previously reported in colon cancer, ovarian tumors and medulloblastoma [11, 15, 16]. Additionally, YAP1 overexpression may be linked to tumor progression resulting in a worse prognosis, as observed in pediatric ACT.

YAP1 plays an important role in cell mechanotransduction, in which its expression represents biochemical signals triggered by mechanical inputs [26, 27]. YAP1 interacts with alpha-catenin binding to cell adhesion molecules and is essential to promote cellular reactions in response to changes in extracellular matrix (ECM) [28]. In a stiff ECM, YAP1 is activated and is accumulated into the nucleus. In contrast, in a soft ECM, YAP1 accumulates in the cytoplasm and is degraded. The increasing ECM rigidity causes a loss of cell junctions leading to epithelial-to-mesenchymal transition (EMT) and metastasis [29]. Additionally, cell-cell junctions are lost in the EMT process, which triggers the Hippo signaling blocking and, consequently, YAP1 activation [26]. In the present study, YAP1 knockdown resulted in a decrease in cell migration and cell viability, demonstrating the potential involvement of YAP1 in adrenocortical cell growth and metastasis. Altogether, these data support the key role of YAP1 in metastasis, which is translated in our data showing an association between increased YAP1 expression and recurrent and/or metastatic disease in pediatric ACT.

Other pathways besides the Hippo signaling can regulate YAP1. Consistent with conserved mechanisms between brain tumorigenesis and development, SHH induces YAP1 expression and its nuclear localization in cerebellar granule neuron precursors, whose proliferation can be driven by YAP1 [15]. We have recently reported that the SHH pathway is downregulated in pediatric ACT [20]. Supporting the idea of a role for YAP1 in ACT pathogenesis, our data showed that *YAP1* is upregulated in pediatric ACT with a poor outcome. Furthermore, YAP1 is linked to the Wnt/beta-catenin pathway, which is the most frequently altered pathway in ACT [3, 30, 31]. In colon cancer, the reduction of YAP1 expression triggered hyperactivity of the Wnt/beta-catenin pathway, supporting the link between Hippo/YAP1 signaling and the Wnt/beta-catenin pathway [18].

In order to evaluate the interaction between YAP1 and the Wnt/beta-catenin pathway, we treated the NCI-H295 ACC cell line with a TCF/beta-catenin complex inhibitor (PNU-74654), which effectively inhibits the Wnt/beta-catenin pathway [21]. The NCI-H295 cell line harbors the p.S45P beta-catenin mutation, which triggers the Wnt/beta-catenin pathway activation. Thus, this cell line is a good model to be used to evaluate the effect of the pharmacological modulation of the Wnt/beta-catenin pathway *in vitro*. Interestingly, we found that inhibition of the Wnt/beta-catenin pathway decreased YAP1 protein expression in parallel to the reduction of beta-catenin expression [21]. Thus, our data support that *YAP1* is a Wnt/beta-catenin target in adrenocortical cells. We observed increased *YAP1* mRNA expression after the inhibition of the Wnt/beta-catenin pathway, which could be explained by post-transcriptional processes, wherein reduced YAP1 protein expression could lead to a negative feedback triggering increased *YAP1* mRNA expression. Similar findings were previously observed in colonic tumorigenesis and tissue renewal. Cai *et al* reported increased YAP1 protein expression in colonic regeneration models, which was not related to increased gene transcription, since *YAP1* mRNA expression was decreased in the intestinal crypts during the regeneration process [32]. On the other hand,*YAP1* silencing resulted in both *CTNNB1*/beta-catenin increase and beta-catenin nuclear expression. These findings support a role of YAP1 in the fine-tuning regulation of the Wnt-beta-catenin pathway in ACC cells. Moreover, it is known that depending on its availability and phosphorylation state, YAP1 can either retain beta-catenin in the cytoplasm or facilitate its transport into the nucleus [33].

Our group had previously described the activation of the Wnt/beta-catenin pathway in pediatric ACT mostly due to underexpression of the Wnt/beta-catenin pathway inhibitors besides *CTNNB1* mutations. Mutations in the beta-catenin gene (*CTNNB1*) are rare in pediatric ACT, but they are associated to a poor prognosis in this group [5]. Therefore, the relationship between the expression of YAP1 and the components of the Wnt/beta-catenin pathway represents a great significance for the enrichment of our results.

YAP1 is the main mediator of the Hippo pathway, which promotes YAP1 downregulation. Other abnormalities in the Hippo pathway could also lead to its activation [34]. Thus, our results offer the prospective to investigate in depth other components of the Hippo pathway such as MST1/2 kinase, SAV regulatory protein and components of the Hippo degradation complex in adrenocortical cells and in ACT. In pediatric ACT, we have not deliberately assessed whether there is a difference in the expression of *YAP1* regarding histology (adenoma and carcinoma) due to the difficulty for histological differentiation in pediatric ACT [35].

In conclusion, our results showed that YAP1 is overexpressed in pediatric ACT with poor outcome. YAP1 plays an important role in adrenocortical tumorigenesis and it is a candidate to be a prognostic marker for pediatric ACT. Therefore, further studies on other cohorts are necessary to support the involvement of the Hippo/YAP1 pathway in adrenocortical tumorigenesis and progression. Moreover, there is a crosstalk between YAP1 and Wnt/beta-catenin pathway in adrenocortical tumorigenesis. Our results open the possibility of future molecular therapies targeting Hippo/YAP1 signaling and linked pathways to treat ACT patients with invasive, recurrent and/or metastatic disease.

## MATERIALS AND METHODS

### Subjects

Forty-two children with ACT diagnosed between 1991 and 2013 at two reference centers in Southeast Brazil (Ribeirao Preto Medical School - University of Sao Paulo and Boldrini Children's Cancer Center, Campinas) were enrolled. Laboratory evaluation, abdominal computed tomography (CT) and/or magnetic resonance imaging (MRI) and patient follow-up were performed as previously described [5]. Disease stage was classified according to the Sandrini classification proposed for pediatric ACTs [9]. Ten adrenal cortices obtained during autopsies from children with non-adrenal endocrine diseases or with Wilm's tumor submitted to surgery (nephrectomy and adrenalectomy) were confirmed to be normal after macroscopic evaluation and used as controls. Fetal adrenal cortices were obtained from spontaneously miscarried fetuses who underwent autopsies in the Department of Pathology, Ribeirao Preto Medical School as described previously were used in the IHQ analysis [20]. This study was approved by the local Ethics Committee (#7534/2010) and a signed statement of informed consent was obtained from the parents of pediatric patients and from the control's relatives.

### RNA isolation and RT-qPCR

Total RNA was extracted using TRIzol® Reagent (Life Technologies) and mRNA was submitted to reverse transcription from 500 ng of total RNA using the High Capacity cDNA Reverse Transcription kit and MultiScribe® enzyme (Life Technologies). For quantitative Real-Time PCR (RT-qPCR), TaqMan® assays (*YAP1*: Hs_00902712_g1 and *CTNNB1:* Hs_00170025_m1, Life Technologies) were used.. Among the three endogenous controls *TBP*, *GUSB* and *GAPDH* initially tested, *GUSB* was the most stably expressed in adrenal tissues. Therefore, for all analyses, mRNA relative expression values were determined by the 2^-ΔΔCt^ method using *GUSB* as endogenous control (4326320E, TaqMan® assay, Life Technologies).

### Immunohistochemical (IHC) analysis

Immunohistochemistry for YAP1 protein was performed in a subset of 35 pediatric ACT samples and 32 fetal adrenals. A mouse monoclonal anti-YAP1 primary antibody (dilution: 1:50; ab56701, Abcam) was used, followed by signal detection with the REVEAL Biotin-Free Polyvalent HRP kit (REVEAL®, Amsbio). Nuclear and/or cytoplasmic labeling was developed with 3,3′-iaminobenzidine (DAB, Vector Laboratories Inc.; brown color) and counterstained with Harris hematoxylin (blue). As negative controls, all specimens were incubated with no primary antibody under identical conditions. All slides were evaluated randomly in at least 10 representative high-power fields (x 400 and x 1000) by an experienced pathologist (author L.Z.R.). These results were blind checked by the authors R.H.A. and S.R.A. YAP1 staining intensity was defined as negative (less than 1%), +1 (weak, 1%–10%), +2 (moderate, 11%–50%), and +3 (strong, > 50%).

### In vitro study

#### NCI-H295 adrenocortical cell line

The NCI-H295 adrenocortical cell line was kindly provided by Professor Claudimara Lotfi (Institute of Biomedical Sciences, University of Sao Paulo) [38]. Cells were cultured as previously described [21]. For all experiments, cell lines were harvested during their third passage. The NCI-H295 cell line was authenticated by analyzing the STR profile as recently described [21].

### Wnt/beta-catenin inhibition

The PNU-74654 compound (Sigma Aldrich), a Wnt/beta-catenin antagonist, was resuspended in dimethyl sulfoxide (DMSO; Sigma Aldrich) at a stock concentration of 31.2 mM, and diluted in complete growth medium to the required concentrations.

NCI-H295 cells were seeded at 2x10^5^ cells per well in 24-well plates for gene expression and protein analysis. After 48 hours, cells were treated with vehicle (0.32% DMSO) and 10, 50 and 100 μM PNU-74654. After 48 hours, cells were harvested for RNA and protein isolation. At least two independent experiments were performed in triplicate.

### siRNA transfection - YAP1 silencing

NCI-H295 cells were seeded in 96, 24 or 6-well cultures plates (2x10^4^, 2x10^5^ or 1x10^6^ cells per well, respectively). After 24 hours, the medium was replaced by antibiotic-free complete medium and the cells were transfected with 25nM of either small-interfering RNA (siYAP1) or a silencer negative control (siNonTargeting) (ON-TARGETplus siRNA, Dharmacon GE) using DharmaFECT 1 transfection reagent (Dharmacon GE). After 24 hours of incubation, the medium was replaced and the cells were harvested after 48 hours for gene expression and immunofluorescence, after 72 hours for transwell migration and after 96 hours for cell viability analysis. *YAP1* silencing experiments were always performed in triplicate.

### Immunofluorescence

NCI-H295 cells (2x10^5^) were seeded on coverslips in 24-well plates 24 hours before siRNA transfection. Forty-eight hours after transfection, the cells were fixed in methanol and blocked with 10% normal horse serum. Beta-catenin was detected using a monoclonal mouse primary anti-B-Catenin antibody (dilution: 1:2000; #610154, BD Biosciences) and a goat anti-mouse IgG1 FITC secondary antibody (dilution: 1:250; sc_2078, Santa Cruz Biotechnology). Nuclear staining was made using DAPi (dilution: 1:25000 #4083, Cell Signaling Technology) and slides were set with Fluoromount (Sigma-Aldrich). Fluorescence was acquired with an Imager.A1 fluorescence microscope (Zeiss) using the AxioVision LE software (Zeiss) with fixed exposure time for all samples.

### Transwell migration assay

Six-transwell insert plates were incubated at 37°C for 12 hours before 1x10^6^ NCI-H295 cells were plated in complete medium. After 24 hours, the cells were transfected in triplicate with siYAP1 or Control as previously described. Twenty-four hours after siRNA incubation, the medium was replaced by serum-free in the inside chamber and by 5% Serum Replacement 3 (Sigma Aldrich) enriched medium in the bottom of the trans-well. After another 48 hours of incubation, the cells were fixed with methanol and non-migrated cells remaining on the topside of the membrane were removed with a cotton swab. The migrated cells were stained with Gills Hematoxylin and images of four representative fields of each insert were taken using an Imager.A1 light microscope (Zeiss) and the AxioVision LE software (Zeiss).

### Cell viability assay

NCI-H295 cells (2x10^4^ per well) were seeded in 96 well plates 24 hours before siRNA transfection as previously described. Ninety-six hours after transfection, 20 μL of Cell Titer 96 Aqueous One Solution (Promega) were added to each well containing 100 μL of complete medium and incubated for 2 hours under normal conditions. Absorbance at 490 nM was obtained using a microplate reader (Bio Rad). Cell viability values were expressed as percentages of control cells. Two independent experiments were performed in triplicate.

### Protein isolation and western blot

Cells were lysed with 100 μL of IP Lysing Buffer (Pierce, Thermo Scientific) and 1μL of Halt Protease and Phosphatase inhibitor cocktail (Thermo Scientific). Protein concentration was measured by the BCA protein assay (Pierce, Thermo Scientific). Equal amounts (20 μg) of protein were subjected to SDS-PAGE, transferred to nitrocellulose membranes, blocked in TBST-T containing 5% skim milk and probed with mouse monoclonal anti-YAP1 primary antibody (dilution: 1:1000; ab56701, Abcam). Anti-GAPDH antibody (dilution: 1:1000, #sc-47724; Santa Cruz Biotechnology) was used as loading control. Complexes were visualized with HRP-conjugated anti-mouse (dilution: 1:4000; #sc-2005; Santa Cruz Biotechnology) antibody and developed by enhanced chemiluminescence (Immun-Star™ WesternC™ Chemiluminescence Kit) on a ChemiDoc XRS+ System (Bio-Rad, Hercules, CA, USA). Acquired bands were analyzed using the Image Lab ™ software (Bio-Rad).

### Statistical analysis

Quantitative variables are reported as median and range and were analyzed by the Mann-Whitney test and by one-way ANOVA with Dunn's post-test. Survival analysis was carried out by Kaplan-Meier curves and compared by the log-rank test, considering death as the unfavorable event and patients who were lost to follow-up were censored considering their last follow-up visit. For *in vitro* analysis, data are described as median and standard error (SEM) and one-way ANOVA followed by Dunnett's multiple comparison tests or Student t-test were used to determine differences between PNU-74654 treatment, *YAP1* silencing, and controls. GraphPad Prism 6.0^®^ software was used for these analyses (GraphPad, San Diego, CA) and the level of significance was set at p≤ 0.05.

In addition, ACT data were adjusted by linear regression based on the Student t-test focused on a Bayesian model. We chose this statistical method due to sample size and problems to use statistical tests based on asymptotic theory. Multiple comparisons were performed by orthogonal contrasts. We used the presence of mutations in the *CTNNB1* gene as an exclusion criterion and therefore pediatric ACT harboring *CTNNB1* mutations were not included in these analyses. Results were analyzed using the OpenBUGS software [36] by estimate observation and 95% credible interval (95% CrI) and therefore there is no p value for them [37].
